# The Native *Hymenoscyphus albidus* and the Invasive *Hymenoscyphus fraxineus* Are Similar in Their Necrotrophic Growth Phase in Ash Leaves

**DOI:** 10.3389/fmicb.2022.892051

**Published:** 2022-05-27

**Authors:** Ari M. Hietala, Ahto Agan, Nina E. Nagy, Isabella Børja, Volkmar Timmermann, Rein Drenkhan, Halvor Solheim

**Affiliations:** ^1^Norwegian Institute of Bioeconomy Research, Steinkjer, Norway; ^2^Institute of Forestry and Engineering, Estonian University of Life Sciences, Tartu, Estonia; ^3^Norwegian Institute of Bioeconomy Research, Ås, Norway

**Keywords:** ash dieback, endophyte, light microscopy, mycobiome, PacBio sequencing, qPCR

## Abstract

The populations of European ash and its harmless fungal associate *Hymenoscyphus albidus* are in decline owing to ash dieback caused by the invasive *Hymenoscyphus fraxineus*, a fungus that in its native range in Asia is a harmless leaf endophyte of local ash species. To clarify the behavior of *H. albidus* and its spatial and temporal niche overlap with the invasive relative, we used light microscopy, fungal species-specific qPCR assays, and PacBio long-read amplicon sequencing of the ITS1-5.8S-ITS2 region to examine fungal growth and species composition in attached leaves of European ash. The plant material was collected from a healthy stand in central Norway, where ash saplings in late autumn showed leaflet vein necrosis like that commonly related to *H. fraxineus*. For reference, leaflet samples were analyzed from stands with epidemic level of ash dieback in southeastern Norway and Estonia. While *H. albidus* was predominant in the necrotic veins in the healthy stand, *H. fraxineus* was predominant in the diseased stands. Otherwise, endophytes with pathogenic potential in the genera *Venturia* (anamorph *Fusicladium*), *Mycosphaerella* (anamorph *Ramularia*), and *Phoma*, and basidiomycetous yeasts formed the core leaflet mycobiome both in the healthy and diseased stands. In necrotic leaf areas with high levels of either *H. albidus* or *H. fraxineus* DNA, one common feature was the high colonization of sclerenchyma and phloem, a region from which the ascomata of both species arise. Our data suggest that *H. albidus* can induce necrosis in ash leaves, but that owing to low infection pressure, this first takes place in tissues weakened by autumn senescence, 1–2 months later in the season than what is characteristic of *H. fraxineus* at an epidemic phase of ash dieback. The most striking difference between these fungi would appear to be the high fecundity of *H. fraxineus*. The adaptation to a host that is phylogenetically closely related to European ash, a tree species with high occurrence frequency in Europe, and the presence of environmental conditions favorable to *H. fraxineus* life cycle completion in most years may enable the build-up of high infection pressure and challenge of leaf defense prior to autumn senescence.

## Introduction

European ash (*Fraxinus excelsior* L.) is threatened by the invasive ascomycete *Hymenoscyphus fraxineus* Baral, Queloz, Hosoya (syn. *H. pseudoalbidus*, anamorph *Chalara fraxinea*) (Kowalski, 2006; Queloz et al., 2011; Baral et al., 2014) considered originating from Asia (Zhao et al., 2013). The current model of the pathogen’s life cycle suggests that ascospores germinating on leaf surfaces give rise to leaf tissue infection, which is followed by mycelial spread through the petiole into shoots and twigs prior to leaf shed, formation of bark lesions in young shoots, and eventually crown dieback (Haňáčková et al., 2017b). Besides the leaf-to-shoot route, also lenticels (Nemesio-Gorriz et al., 2019) and direct penetration of the epidermis of young shoots (Mansfield et al., 2019) may offer additional entrances for the pathogen to stem tissues. The disease symptoms include local necroses of leaf tissues, particularly in vascular tissue areas, wilting of leaves and young shoots, shoot dieback, premature leaf fall, and variously colored bark necroses of variable size (Przybył, 2002).

The high propagule pressure of *H. fraxineus* is typical of invasive species (Cross et al., 2017). Coupled with the continental scale dieback of European ash, the pathogen poses a set of cascading impacts on the biodiversity associated with this keystone tree species in Europe (Pautasso et al., 2013; Mitchell et al., 2014). It has been predicted that ash dieback represents an insidious threat to sessile affiliate communities like lichens (Jönsson and Thor, 2012). This scenario would appear to concern *H. albidus* (Roberge ex Gillet) W. Phillips, a native ash-foliage specialized species that has declined since the arrival of its relative, *H. fraxineus* (McKinney et al., 2012; Hietala et al., 2018b). *H. albidus* is regarded as a relatively rare species based on the limited number of herbaria deposits (Baral and Bemmann, 2014; Drenkhan et al., 2016).

Both *H. albidus* and *H. fraxineus* use the leaf vein system as a sporulation substrate during the saprotrophic phase in leaf debris. As recently reviewed (Hietala et al., 2018a), there are no striking differences between *H. albidus* and *H. fraxineus* when it comes to their sporulation regime, toxin production, and enzymatic repertoire. The life cycle of *H. albidus* has been subject to speculation as the species is known only from the saprotrophic phase (Baral and Bemmann, 2014). Based on observations of mostly herbaria material, Baral and Bemmann (2014) concluded that the pseudosclerotial plates involved in securing the saprotrophic niche are relatively small for *H. albidus*, whereas those of *H. fraxineus* typically extend throughout the entire petiole and rachis system. In consistent with an obviously high saprobic competence, both *Hymenoscyphus* species harbor an extensive repertoire of cell wall carbohydrate active enzymes and appear better equipped for saprobic feeding than necrotrophic members of Helotiales with characterized genomes (Stenlid et al., 2017). In inoculation trials applying tissue wounding, *H. albidus* induced shorter lesions in the rachis of European ash and American green ash (*F. pennsylvanica*) in comparison to those induced by *H. fraxineus* (Kowalski et al., 2015). Similarly, *H. albidus* inoculations on stem wounds of European ash, American green ash, and Manchurian ash (*F. mandshurica*) have induced only short lesions (Gross and Holdenrieder, 2015; Kowalski et al., 2015; Gross and Sieber, 2016).

In the present study, we examined the fungal composition and hyphal growth pattern in attached leaves of European ash in a stand located slightly north of what was considered the ash dieback frontier the previous year (Laforest-Lapointe et al., 2017). No shoot dieback symptoms were present in the stand, but some saplings showed leaflet vein necrosis similar to that associated with *H. fraxineus*. We originally assumed these symptoms would be the first sign that this forest was infested by *H. fraxineus* – instead, the lesions turned out to be associated with *H. albidus*. Thus, the aim of the study was to clarify the nature of the association of *H. albidus* with living leaf tissues of European ash and compare that with the behavior of *H. fraxineus*. As an additional reference, we used the structure of fungal communities associated with ash leaflets in stands with epidemic level of ash dieback in southeastern Norway and Estonia.

## Materials and Methods

### Study Sites and Sampling

**FIGURE 1:**
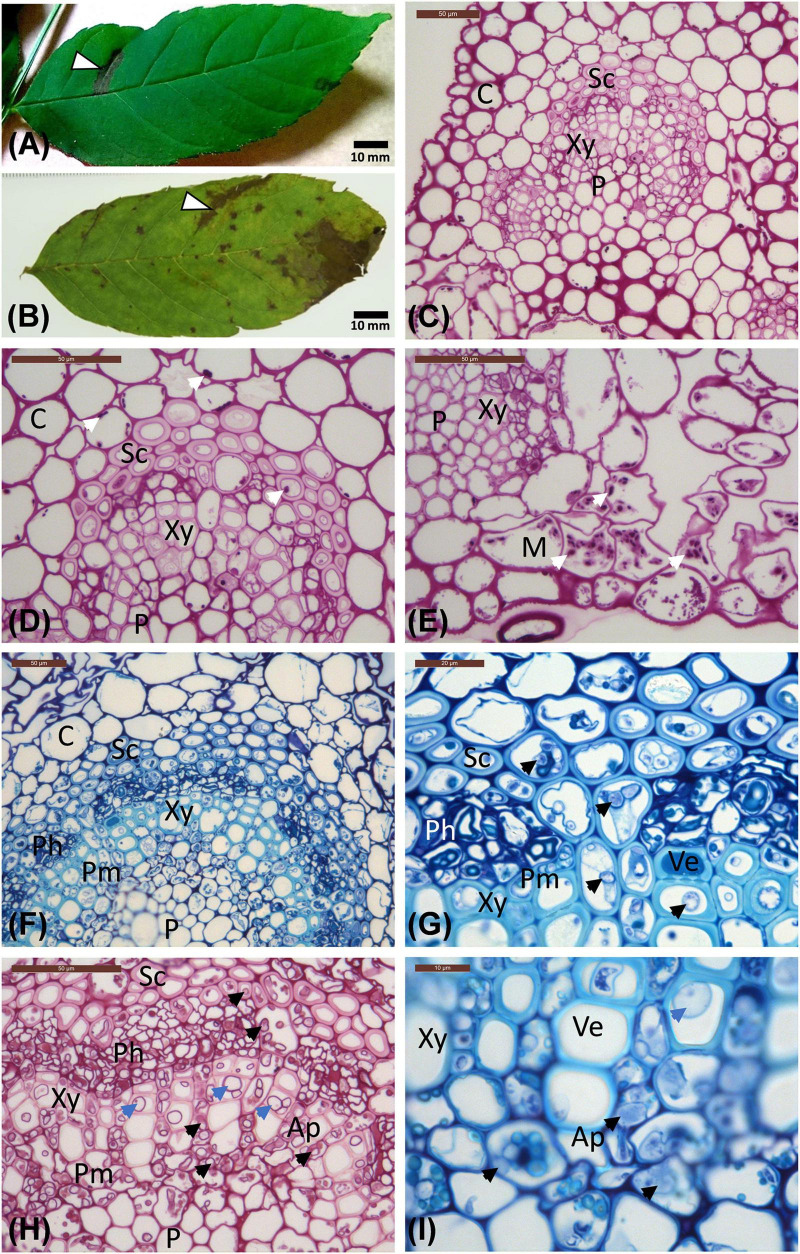
Leaflet images and micrographs of transverse sections of leaflet veins. **(A)** Leaflet from the Stjørdal site, with a necrotic lesion along one side vein (arrowhead, sample S1) in an otherwise healthy appearing leaflet. **(B)** Leaflet from the Norderås site with a broad necrotic lesion following one side vein, and several small lesions mostly in leaf blade areas. **(C–E)** Transverse sections of veins from asymptomatic regions of leaflets from Stjørdal **(C,D)** and Norderås **(E)** show the organization of different cell types and the presence of some purple-red stained starch grains (white arrows). **(F–I)** Transverse sections of veins from lesion areas in leaflets sampled from Stjørdal **(F,G)** or Norderås **(H,I)** and showing hyphal presence in sclerenchyma, phloem, axial parenchyma, and perimedullary pith (examples pointed with black arrowheads) and formation of tyloses in vessel elements (examples pointed with blue arrowheads). Note also the appressoria-like swelling upon hyphal spread between two cells of axial parenchyma **(I)**. Ap, Axial parenchyma; C, Cortex; M, Mesophyll; Ph, Phloem; P, Pith; Pm, Perimedullary parenchyma; Sc, Sclerenchyma; Ve, Vessel elements; Xy, xylem.

Sampling was carried out in a natural forest stand (63°26′46.84806″N, 10°59′7.71109″E) located on a north–west facing steep slope in central Norway in the municipality of Stjørdal (referred to as Stjørdal site in the text). Ash is the canopy-forming tree species in the stand, the dominant trees being up to 20 m tall. In addition, ash is present in the understory along with some saplings of rowan (*Sorbus aucuparia* L.), aspen (*Populus tremula* L.), bird cherry (*Prunus padus* L.), alder (*Alnus* spp.), and willow (*Salix* spp.). We have collected *H. albidus* ascomata from this stand in 2013 and 2017 (unpublished data). In 2016, the stand was visited once a week throughout the season to observe putative symptoms related to *H. fraxineus* since the ash dieback frontier in 2015 (Solheim and Hietala, 2017) was so close to the subjected stand that we expected the pathogen to reach the site as early as in 2016. The first symptomatic leaves, with necrotic lesions associated with leaflet veins, were observed 01.10.2016, and four compound leaves with necrotic lesions typically present only in one leaflet per compound leaf were collected (Figure 1A and Supplementary Figure 1). In the following year, the stand was visited once a week and five compound leaves with necrotic lesions on leaflet veins were collected from saplings on the day of their first observation, 22.09.2017.

Since the necrotic leaflet lesions turned out to host *H. albidus* instead of *H. fraxineus*, for reference purposes, European ash leaflets with necrotic lesions in leaflet vein areas were collected on 22.09.2017 from saplings in an ash stand in southeastern Norway (Ås municipality, 59°40′44′′N, 10°46′31′′E, 100 m a.s.l, referred to as Norderås site in the text). As an additional reference, we used the structure of fungal communities associated with ash leaflets in the above-mentioned Norderås site and in an ash stand located in Vedu village (referred to as Vedu site in the text) in southeastern Estonia (Estonia 58°29′06.4′′N, 26°45′25.2′′E, 100 m a.s.l), these data being generated from ash leaflets collected in September 2014 in our previous metagenomics study (Agan et al., 2020). Both reference stands showed epidemic level of ash dieback. The Norderås site, subjected to several prior studies of ash dieback (Timmermann et al., 2011; Hietala et al., 2013; Agan et al., 2020), is a naturally regenerated moist forest with rich understory vegetation dominated by meadowsweet [*Filipendula ulmaria* (L.) Maxim.]. European ash is present both as a canopy-forming tree (the largest ash trees ranging between 20 and 27 m in height) and in the understory, along with rowan, aspen, bird cherry, and downy birch (*Betula pubescens* Ehrh.), alder and willows. The Vedu site is mostly dominated by *F. excelsior*, followed by common oak (*Quercus robur* L.) and Norway maple (*Acer platanoides* L.), with occurrence frequencies of 85, 10, and 5%, respectively. The understory is mostly comprised of ash, rowan, Norway maple, some old apple trees (*Malus domestica* Borkh. nom. illeg.), and currants (*Ribes* spp.).

### Light Microscopy and Staining

Tissue pieces, ca. 4 mm^2^ in size, were dissected with a surgeon knife from necrotic lesions centered on leaflet veins, and from corresponding green tissues from five symptomatic leaflets collected from five saplings per site (Figures 1A,B and Supplementary Figure 1). The dissected subsamples were stored in a fixative [3 ml of 2% paraformaldehyde and 1.25% glutaraldehyde in 50 mM L-piperazine-N-N’-bis (2-ethane sulfonic) acid buffer (pH 7.2)] at 4°C prior to processing and embedding in L. R. White resin (TAAB, Aldermaston, Berkshire, England), according to Nagy et al. (2000). Semi-thin cross sections (1.5 μm) were cut by an LKB 2128 Ultratome (Leica Microsystems, Wetzlar, Germany), and dried onto glass slides. Sections used for routine observations were stained with Stevenel’s blue (del Cerro et al., 1980). Since *H. fraxineus* shows a high affinity to starch-rich cells in ash shoots (Matsiakh et al., 2016), Periodic acid-Schiff (PAS; Sigma, St. Louis, MO, United States) staining was used to detect starch grains and carbohydrate-rich compounds (Hotchkiss, 1948; McManus, 1948). Since ash leaves infected by *H. fraxineus* show an accumulation of phenolics (Nielsen et al., 2022), unstained sections were examined with autofluorescence (natural emission) for detection of cellular polyphenolic components as described by Franceschi et al. (1998), using a Leica DMR light microscope operated in epifluorescence mode. Blue light (450–490 nm) and a long-band-pass filter (>520 nm) were used for excitation and visualization of polyphenolics, respectively. The presence of starch and fungal hyphae within axial parenchyma cells of the vascular tissue was estimated for each sample from three randomly chosen microscopic fields by using the following grading: absent (0), some cells showed the feature (1), several cells showed the feature (2), most cells showed the feature (3).

### DNA Isolation

Subsamples with a size of 12 ± 3 mm^2^ were dissected with a sterile surgeon knife from representative lesion areas and corresponding green tissues from four symptomatic leaflets collected in 2016 and five symptomatic leaflets collected in 2017 at the site in Stjørdal (Figure 1A and Supplementary Figures 1A–G), and from five symptomatic leaflets collected in 2017 at the Norderås site (Figure 1B and Supplementary Figures 1H–L), each leaflet originated from a different sapling.

### Real-Time Polymerase Chain Reaction

For detection of *H. fraxineus*, we used the forward primer Cfrax-F 5′-ATTATATTGTTGCTTTAGCAGGTC-3, reverse primer Cfrax-R 5′-TCCTCTAGCAGGCACAGTC-3′ and probe C-frax-P5′-FAM- CTCTGGGCGTCGGCCTCG-BHQ1-3′ designed and tested for species specificity by Ioos et al. (2009). For detection of *H. albidus*, we used the primer and probe set designed and tested for species specificity by Husson et al. (2011), with the modification of using JOE as the reporter dye (Hietala et al., 2013) instead of YY: forward primer Halb-F 5′-TATATTGTTGCTTTAGCAGGTCGC-3′, reverse primer Halb-R 5′-ATCCTCTAGCAGGCACGGTC-3′, and probe Halb-P5′-JOE-CCGGGGCGTTGGCCTCG-BHQ1-3′. For quantification of *H. fraxineus* DNA, the primer and probe concentrations were 300 and 100 nM, respectively, as described by Ioos et al. (2009). For quantification of *H. albidus* DNA, 900 nM concentrations were used for the primers and the probe (Hietala et al., 2013).

Amplification was performed in Takyon™ Low Rox Probe MasterMix dTTP Blue (Eurogentec, Seraing, Belgium) according to manufacturer instructions with the Applied Biosystems ViiA 7 qPCR machine. For the assays of both *H. fraxineus* and *H. albidus*, polymerase chain reaction (PCR) cycling parameters were 95°C for 10 min, followed by 40 cycles of 95°C for 15 s and 65°C for 55 s. To ensure that the cycle threshold values from the experimental samples fell within the standard curves and to investigate the presence of compounds inhibitory to PCR, 3-log dilution series were prepared for all the experimental samples. Each experimental sample had undiluted DNA as the most concentrated, and all log dilutions of a sample were used as templates in real-time PCR. Three μL of the DNA solution were used as the template for each 25-μl PCR reaction. Each reaction was repeated 2 times. Standard curves for DNA quantity were constructed based on DNA extracted from pure cultures of *H. fraxineus and H. albidus*. The obtained Ct values were plotted against log-transformed template DNA amounts to prepare a standard curve to estimate pathogen DNA by interpolation in leaflet samples.

### High Throughput Sequencing of the ITS rDNA Gene Cluster

DNA from the samples collected from Stjørdal in 2016 were subjected to fungal community profiling. For this purpose, primers ITS4ngs (Tedersoo et al., 2014) and ITS1catta (Tedersoo et al., 2019) were used to amplify fungal DNA. The PCR products were sequenced using PacBio platform at the University of Oslo, Norway. PacBio has been recently used successfully in metabarcoding analysis of microorganisms in different plant, tree, and insect species, with the lesson that the long DNA barcodes (500–1500 bp) enabled by the platform substantially improve the resolution in species identification (Loit et al., 2019; Tedersoo and Anslan, 2019; Tedersoo et al., 2019; Agan et al., 2020, 2021; Jürisoo et al., 2021). The primer ITS1catta was originally designed to differentiate *H. albidus* from *H. fraxineus*, and to avoid amplification of plant DNA and the long intron in the 3′ end of the rRNA 18S gene of *Hymenoscyphus* species (see Tedersoo and Anslan, 2019; Agan et al., 2020). Primer ITS4ngs was equipped with 10–12 base multiplex identifier (MID) indices that differed from any other indices by at least four bases.

Conventional PCR was carried out with two replicates for each sample in 25-μl-reaction volumes according to Agan et al. (2020). The PCR reactions were checked for the presence of a product on 1% agarose gels. In case of no visible band, we repeated the amplification by increasing the number of cycles up to 35. The PCR products were purified using GeneJET DNA purification kit (Thermo Fisher Scientific, Waltham, MA, United States) following the manufacturer’s instructions.

The amplicons were pooled into one sequencing library on an equimolar basis. Library preparation followed the protocols established for the RS II instrument of PacBio third-generation sequencing platform (Pacific Biosciences, Inc., Menlo Park, CA, United States). The diffusion method was used when loading samples to SMRT cells. Sequencing was performed using P6-C4 chemistry for 10 h following Tedersoo et al. (2018).

### Bioinformatics Analysis

Bioinformatics was carried out according to Agan et al. (2020), using various programs implemented in PipeCraft 1.0 (Anslan et al., 2017). Using Mothur (Schloss et al., 2009), reads <100 bp were removed and the longer sequences were demultiplexed, allowing 1-base differences to index and 2-base differences to primer. Using UCHIME (Edgar et al., 2011), *de novo* chimera filtering was performed. The full-length Internal Transcribed Spacer (ITS) region was extracted from the rRNA genes using ITSx (Bengtsson-Palme et al., 2013). Using CD-HIT (Fu et al., 2012), sequences were clustered into Operational Taxonomic Units (OTUs) based on 99% sequence similarity to ensure differentiation between *H. albidus* and *H. fraxineus*. The remaining OTUs were taxonomically identified based on a comparison of representative sequences against the UNITE v. 9 database (Kõljalg et al., 2013). OTUs were considered as members of Fungi if their representative sequences matched the best fungal taxa at *e*-value < e^–50^. Representative sequences that had >97% sequence similarity to reference sequences were assigned to species hypotheses (SHs) based on UNITE (Kõljalg et al., 2013). Higher level classification of Fungi was based on the *e*-value and sequence similarity criteria of Tedersoo et al. (2014).

### Statistical Analysis

PAST3 (Hammer et al., 2001) was used for OTU richness calculation for each sample and for rarefaction to check if the number of sequences was sufficient to capture most of the species diversity. A possible effect of leaf symptoms (i.e., necrotic lesion vs. green tissue) in the Stjørdal site was, due to a low number of samples (10), tested using ANOVA with Tukey’s *post hoc* test. Differences between symptomatic and healthy tissues were considered significant with *p* ≤ 0.05.

For reference purposes, concerning fungi associated with ash leaflets in stands with the epidemic level of ash dieback, we used the mycobiome data retrieved from our recent study (Agan et al., 2020). These data were obtained from leaflets of symptomatic European ash saplings collected in September 2014 from the Norderås and Vedu sites described above (see section Study Sites and Sampling) and processed with the same sequencing and sequence processing pipeline as in the current study.

To test for differences in fungal communities between the Stjørdal, Norderås, and Vedu sites, we used PERMANOVA+ (Anderson et al., 2008). OTU abundance matrix was square root transformed to reduce the effect of dominant species. Bray-Curtis similarity (Bray and Curtis, 1957) was used as a distance measure. The fungal community structure was visualized using PCO as implemented in Primer v6 (Clarke and Gorley, 2006).

## Results

### *Hymenoscyphus albidus* and *H. fraxineus* DNA Levels in Leaflet Tissues at the Studied Sites

The five samples from necrotic vein areas from the four symptomatic leaflets collected in 2016 from Stjørdal were all positive for *H. albidus* and negative for *H. fraxineus* DNA-specific qPCR assay. The DNA amount estimated for *H. albidus* ranged between 1.6 and 251.7 pg per mm^2^ leaflet area (Table 1). None of the five samples from asymptomatic tissues of these leaflets were positive for *H. albidus* or *H. fraxineus*. Five of the six samples collected in 2017 from necrotic vein areas of the five symptomatic leaflets were positive for *H. albidus* and negative for *H. fraxineus* DNA specific qPCR assay – the DNA amount detected for *H. albidus* ranged between 1.6 and 179.6 pg per mm^2^ leaflet area (Table 1). Of the six samples from asymptomatic regions from these leaflets, five were negative for *H. albidus* and *H. fraxineus*, and one was positive for *H. albidus* (62.7 pg DNA per mm^2^).

**TABLE 1:** Summary of data from qPCR and microscopy analyses of necrotic and green vein tissues of leaflets collected from the Stjørdal and Norderås sites (n.e., not examined).

Sample code^#^	Photo	Date of sampling	qPCR (pg DNA/mm^2^)		PacBio sequencing	Microscopy*	
			*H. albidus*	*H. fraxineus*	Dominant species	Starch	Hyphae
S1.L1	Supplementary Figure 1A	01.10.2016	251.7	0	*H. albidus*	1	2
S1.G1	Supplementary Figure 1A	01.10.2016	0.0	0	*B. crocea*	3	0
S2.L1	Supplementary Figure 1B	01.10.2016	208.4	0	*H. albidus*	1	2
S2.G1	Supplementary Figure 1B	01.10.2016	0.0	0	*B. crocea*	0	0
S3.L1	–	01.10.2016	6.4	0	*V. fraxini*	n.e.	n.e.
S3.G1	–	01.10.2016	0.0	0	*V. fraxini*	n.e.	n.e.
S3.L2	–	01.10.2016	51.0	0	*H. albidus*	n.e.	n.e.
S3.G2	–	01.10.2016	0.0	0	*Vishniacozyma* spp.	n.e.	n.e.
S4.L1	–	01.10.2016	1.6	0	Few sequences	n.e.	n.e.
S4.G1	–	01.10.2016	0.0	0	*V. fraxini*	n.e.	n.e.
S5.L1	Supplementary Figure 1C	22.09.2017	0	0	n.e.	0	0
S5.G1	Supplementary Figure 1C	22.09.2017	0	0	n.e.	1	0
S6.L1	Supplementary Figure 1D	22.09.2017	29.0	0	n.e.	1	0
S6.G1	Supplementary Figure 1D	22.09.2017	0.0	0	n.e.	1	0
S7.L1	Supplementary Figure 1E	22.09.2017	1.6	0	n.e.	2	1
S7.G1	Supplementary Figure 1E	22.09.2017	0.0	0	n.e.	1	0
S7.L2	Supplementary Figure 1E	22.09.2017	19.0	0	n.e.	2	3
S7.G2	Supplementary Figure 1E	22.09.2017	0.0	0	n.e.	3	0
S8.L1	Supplementary Figure 1F	22.09.2017	179.6	0	n.e.	1	3
S8.G1	Supplementary Figure 1F	22.09.2017	62.7	0	n.e.	2	1
S9.L1	Supplementary Figure 1G	22.09.2017	18.4	0	n.e.	1	0
S9.G1	Supplementary Figure 1G	22.09.2017	0.0	0	n.e.	1	0
N1.L1	Supplementary Figure 1H	22.09.2017	n.e.	223.8	n.e.	2	2
N1.G1	Supplementary Figure 1H	22.09.2017	n.e.	0	n.e.	2	0
N1.L2	Supplementary Figure 1H	22.09.2017	n.e.	250.0	n.e.	2	3
N1.L/G2	Supplementary Figure 1H	22.09.2017	n.e.	42.4	n.e.	1	3
N2.L1	Supplementary Figure 1I	22.09.2017	n.e.	40.4	n.e.	2	3
N2.G1	Supplementary Figure 1I	22.09.2017	n.e.	0	n.e.	2	0
N2.L2	Supplementary Figure 1I	22.09.2017	n.e.	25.8	n.e.	2	3
N2.G2	Supplementary Figure 1I	22.09.2017	n.e.	14.1	n.e.	1	2
N3.L1	Supplementary Figure 1J	22.09.2017	n.e.	254.1	n.e.	1	3
N3.L2	Supplementary Figure 1J	22.09.2017	n.e.	281.6	n.e.	1	3
N3.L3	Supplementary Figure 1J	22.09.2017	n.e.	122.6	n.e.	2	3
N3.G1	Supplementary Figure 1J	22.09.2017	n.e.	322.7	n.e.	3	0
N4.L1	Supplementary Figure 1K	22.09.2017	n.e.	4.7	n.e.	2	3
N4.G1	Supplementary Figure 1K	22.09.2017	n.e.	0	n.e.	1	0
N5.L1	Supplementary Figure 1L	22.09.2017	n.e.	1.3	n.e.	1	3
N5.G1	Supplementary Figure 1L	22.09.2017	n.e.	0	n.e.	2	0
N5.L2	Supplementary Figure 1L	22.09.2017	n.e.	22.5	n.e.	0	3

*#S, Stjørdal; N, Norderås; L, necrotic lesion; G, green tissue.*

*Frequency of presence within axial parenchyma of the vascular cylinder: 0 = absent; 1 = present in some cells; 2 = present in several cells; 3 = present in most cells.

The 10 subsamples from necrotic vein areas of the five symptomatic leaflets collected in Norderås were all positive in *H. fraxineus* DNA specific qPCR assay. The extrapolated DNA amount of *H. fraxineus* ranged between 1.3 and 254.1 pg per mm^2^. Four of the six samples from asymptomatic tissue were negative for *H. fraxineus* while the remaining two showed 14.1 and 322.7 pg per mm^2^ of *H. fraxineus* DNA. These samples were not profiled by *H. albidus* qPCR as our previous fungal metabarcoding study on ash leaflets collected in 2014 (Agan et al., 2020) indicated that this fungus was no longer present in the stand.

### Histological Observations From Necrotic and Green Leaflet Tissues

#### Stjørdal Site

**FIGURE 2:**
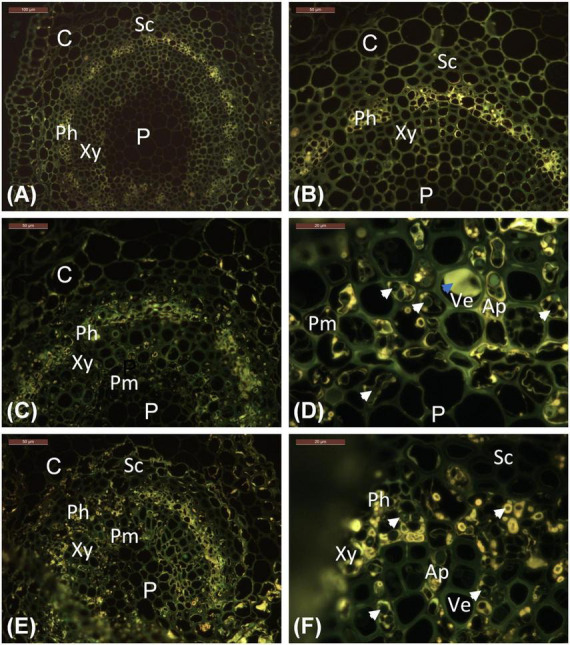
Location of phenolics in leaflet veins, as indicated by bright yellow auto-fluorescence characteristic of polyphenolic compounds. **(A,B)** Stjørdal, asymptomatic vein. **(C,D)** Stjørdal, lesion area in a vein. **(E,F)** Norderås, lesion area in a vein. Examples of hyphae growing in axial parenchyma with phenolic deposits are indicated by white arrowheads. One example of tylosis with a vessel element is indicated by a blue arrowhead. Ap, Axial parenchyma; C, Cortex; Ph, Phloem; P, Pith; Pm, Perimedullary parenchyma; Sc, Sclerenchyma; Ve, Vessel elements; Xy, xylem.

The cell types of ash referred to when describing the histological observations are indicated in Figure 1C. The amount of starch, visualized by PAS staining, varied considerably across samples. Some asymptomatic regions contained relatively much starch, some almost none. Similarly, some symptomatic regions contained a moderate level of starch, some practically none. Within the vascular cylinder of leaflet veins, some starch was present within the phloem and the axial parenchyma surrounding vessel elements (Figure 1D). The highest starch levels were generally observed within the palisade and spongy parenchyma associated with asymptomatic leaflet blade regions (Figure 1E). Tyloses or gum formation within vessel elements were not observed in asymptomatic leaf areas. In asymptomatic regions, polyphenolic droplets, based on blue light autofluorescence detection, were occasionally present in phloem cells and in some axial parenchyma cells aligning xylem vessels and showing a thin polyphenol coating on the cell wall (Figures 2A,B). Samples from asymptomatic regions showed occasionally unidentified fungal propagules on the leaflet surface and within the epidermis, but no fungal hyphae were observed deeper in the tissues.

Within necrotic leaflet areas, there were visually striking differences between samples in the presence of fungal hyphae. While no attempts were made to obtain any quantitative estimates, hyphae of unidentified fungi were particularly abundant in the samples S1.L1, S2.L1, and S8.L1 from necrotic vein areas with the highest level of *H. albidus* DNA. The hyphae, generally with a diameter of 2–6 μm, were most prolific within sclerenchyma, phloem, and the axial parenchyma surrounding vessel elements (Figures 1F,G). While hyphae were primarily spreading in the axial direction in plant tissues, lateral growth of fungal hyphae was occasionally seen in collenchyma and cortex parenchyma associated with veins, and in palisade parenchyma of leaflet blade. Based on blue light autofluorescence detection, phenolic substances, present either as droplets or as a coating of the cell wall, were especially pronounced in phloem and the axial parenchyma in necrotic vein regions in comparison to uncolonized regions (Figures 2C,D). In the necrotic regions, hyphae were frequently surrounded by a phenolic substance (Figure 2D).

#### Norderås Site

In most respects, the histological observations were similar to those recorded for the material from the Stjørdal site. Hyphae with a diameter of 3–6 μm were prolific in sclerenchyma, phloem, and axial parenchyma (Figures 1H,I) of the samples where the highest level of *H. fraxineus* DNA was detected. An exception was the asymptomatic sample N3.G1, taken from a side vein right next to a necrotic leaflet midvein, which showed a high *H. fraxineus* DNA amount. The corresponding N3.G1 sample used for microscopy, taken further away from the necrotic midvein, showed no presence of hyphae. The presence of starch was generally infrequent but most prominent in palisade and spongy mesophyll of the leaf blade in green tissues (Figure 1E). Vessel-associated tyloses (Figures 1H,I) and occasional gum deposition were observed in necrotic leaflet vein areas. Irregular appressoria-like swellings and narrow penetration hyphae were observed in connection with lateral hyphal spread between axial parenchyma cells (Figure 1I). Phenol-coated fungal propagules were common in phloem and parenchyma cells (Figures 2E,F). On a visual basis, the intensity of autofluorescence associated with phenols appeared generally more pronounced in necrotic leaflet vein areas in comparison to leaflets colonized by *H. albidus* in Stjørdal.

### Fungal Species Composition in Stjørdal

**FIGURE 3:**
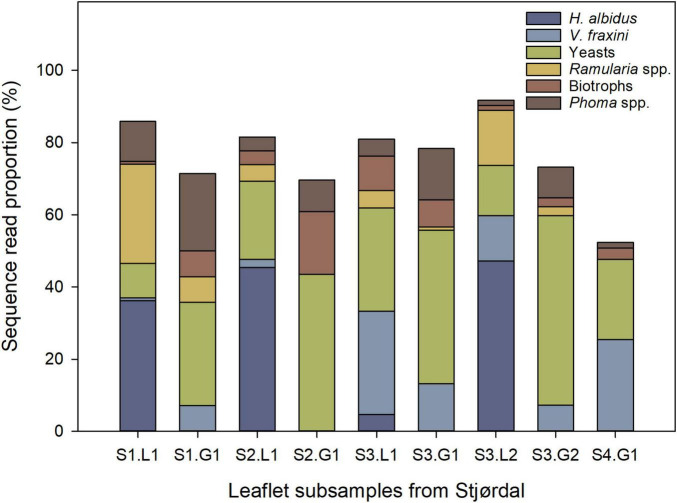
Relative abundance of *H. albidus* and other prominent species or functional groups in four ash leaflets from four different ash saplings from the Stjørdal site (S), L referring to lesion area, and G to asymptomatic green tissue. The sample S4.L1 was excluded owing to the low sequence read count. The number following S refers to the leaflet number, and the number following L or G refers to subsample number.

The relative sequence read abundances of *H. albidus* and other major species or functional groups at the Stjørdal site are shown in Figure 3. *Hymenoscyphus albidus* was present in four out of the five samples from necrotic lesions – it accounted for 38.8% of all sequences in these samples (only a total of three sequences were obtained from the deviating sample S4.L1), but was absent in the corresponding samples from green tissues.

No other fungus than *H. albidus* separated the lesion and asymptomatic samples, i.e., all the other fungi were detected in both types of samples. Of the other prominent species, *Ramularia* spp. showed higher sequence read proportions in lesions than in green tissues. Unidentified species of *Phoma* were present in both lesion and green-tissue samples, with a tendency of the latter to show the higher relative abundance of these species. Yeasts, primarily in the genera *Bullera*, *Dioszegia*, and *Vishniacozyma*, and biotrophic fungi (*Exobasidium* and *Taphrina* species) had a higher relative abundance in green tissues in comparison to lesions. No tissue type pattern was observed in the relative abundance of *Venturia fraxini*. Excluding *H. albidus*, no statistically significant (*p* > 0.05) differences in relative abundances of the 15 most prevalent species occurred between lesions and green areas in the Stjørdal site, possibly due to the small sample size.

Based on the literature, the detected OTUs were divided into three functional groups: fungi that can occur as endophytes but also have the capacity to cause necrosis (endophyte/necrotroph), biotrophic pathogens, and fungi than are not pathogenic and can occur both as epiphytes and as endophytes (epiphyte/endophyte). The functional group assigned to each OTU is shown in Supplementary Table 1. When comparing relative abundances of fungal functional groups between green leaf areas and areas with lesions in the samples from Stjørdal, only the group endophyte/necrotroph showed a slight, but significant difference, with lesion areas having a higher relative abundance of these fungi than green areas (*p* < 0.05). The group epiphyte/endophyte showed a trend toward higher relative abundance in green leaf areas compared to lesion areas (*p* = 0.06), whereas no such difference was observed for biotrophic fungi (*p* = 0.43).

### Comparison of Fungal Species Composition Between Stjørdal, Norderås, and Vedu

After removing low quality sequences and singletons, the final dataset containing the data from all the three sites for 28 samples included 5,849 full ITS sequences that represented 240 OTUs. The ten samples from Stjørdal generated 649 sequences and 80 OTUs, the 10 samples from Norderås 2505 sequences and 150 OTUs, and the eight samples from Vedu 2,695 sequences and 144 OTUs. Of these, 63% were assigned as Ascomycota, 28% as Basidiomycota, and 8.9% as Fungi. Rarefaction analysis of sequences from the Stjørdal, Norderås, and Vedu sites revealed on average (mean ± SE) 19 ± 3.27, 52.3 ± 2.8, and 30.5 ± 6.0 fungal OTUs per sample, respectively. No significant difference in fungal species richness between symptomatic leaf areas and green areas was found in the Stjørdal site.

**FIGURE 4:**
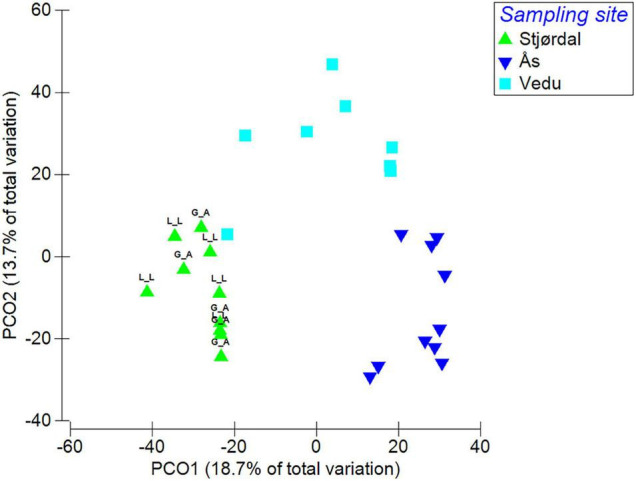
Species composition differences between the three sites (L_L refers to subsamples taken from leaflet lesion area and G_A to subsamples from green leaflet area). Data were square root transformed and resemblance calculated by S17 Bray-Curtis similarity.

The PERMANOVA analysis showed significant differences in fungal species composition (*p* < 0.01) between the three sites. Site accounted for 32.2% of fungal composition differences within the dataset. Differences between sites are visualized in a PCO plot, where the two axis accounted for 32.4% of the total variation in the dataset (Figure 4).

**FIGURE 5:**
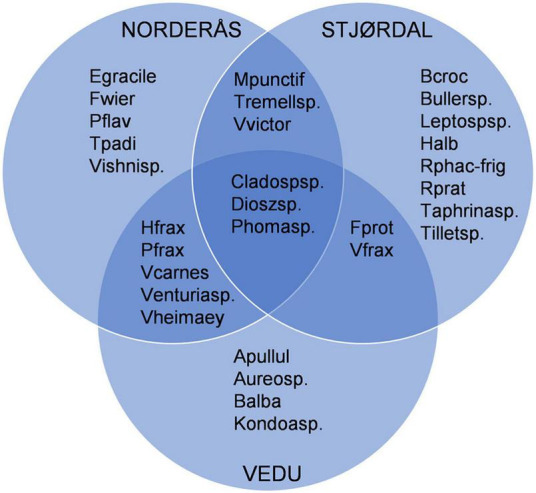
Overview of the 20 most common OTUs that are shared or unique to each site, OTUs that were identified only as Fungi sp. were excluded from the diagram. Explanation: Apullul, *Aureobasidium pullulans*; Aureosp., *Aureobasidium* sp.; Balba, *Bullera alba*; Bcroc, *Bullera crocea*; Bullersp, *Bullera* sp.; Cladospsp., *Cladosporium* sp.; Dioszsp., *Dioszegia* sp.; Egracile, *Exobasidium gracile*; Fwier, *Filobasidium wieringae*; Fprot, *Fusicladium proteae*; Halb, *Hymenoscyphus albidus*; Hfrax, *Hymenoscyphus fraxineus*; Kondoasp., *Kondoa* sp.; Leptospsp., *Leptosphaeriaceae* sp.; Mpunctif, *Mycosphaerella punctiformis*; Pflav, *Papiliotrema flavescens*; Pfrax, *Phyllactinia fraxini*; Phomasp., *Phoma* sp.; Rphac-frig, *Ramularia phacae-frigidae*; Rprat, *Ramularia pratensis*; Tpadi, *Taphrina padi*; Taphrinasp., *Taphrina* sp.; Tremellsp*., Tremellomycetes sp*.; Tilletsp., *Tilletiopsis* sp.; Vfrax, *Venturia fraxini*; Venturiasp*., Venturia* sp.; Vcarnes, *Vishniacozyma carnescens*; Vheimaey, *Vishniacozyma heimaeyensis*; Vishnisp., *Vishniacozyma* sp.; Vvictor, *Vishniacozyma victoriae*.

The most abundant OTUs identified at the species or genus level and their presence in each site are shown in Figure 5. In the Stjørdal site, the 20 most abundant OTUs accounted for 78.3% of all sequences obtained from this site, *H. albidus* being the most abundant with a sequence read percentage of 21.6%. The 20 most common OTUs in Norderås accounted for 73% of all sequences obtained from this site. *H. fraxineus* was the most abundant species with a relative abundance of 20%. In the Vedu site, the 20 most abundant OTUs accounted for 83.6% of all sequences from samples in this site, the most abundant species being *H. fraxineus* with a relative abundance of 29.4%. The fungal genera *Phoma*, *Venturia* (and associated *Fusicladium* anamorph), *Mycosphaerella* (and associated *Ramularia* anamorphs), and *Cladosporium* with endophytic and/or pathogenic stages in their life cycle were common to all sites and constituted 24.3, 9.1, and 34.2% of the sequences in Stjørdal, Norderås, and Vedu, respectively. Yeasts, primarily in genera *Vishniacozyma*, *Dioszegia*, and *Bullera*, constituted 20.3, 27.1, and 10.6% of the sequences in Stjørdal, Norderås, and Vedu, respectively. Biotrophic fungi in the genera *Taphrina* (common in Stjørdal and Norderås), *Phyllactinia* (common in Norderås and Vedu), and *Exobasidium* (common in Norderås) constituted 1.4, 11.8, and 1.2% of the sequences in Stjørdal, Norderås, and Vedu, respectively.

## Discussion

### Relation Between *Hymenoscyphus* Growth Mode, Infection Pressure, and Tissue Condition

While the association of *H. fraxineus* with necrotic lesions on leaf veins has been confirmed by inoculation studies (Nielsen et al., 2017), it has been a subject of discussion whether *H. albidus* could infect living leaves of European ash. Baral and Bemmann (2014) wrote that “it can be assumed that the ascospores (of *H. albidus*) infect living leaves, and the mycelium grows endophytically inside them, similar as it is known in *H. fraxineus*.” As a response, Kowalski et al. (2015) wrote: “*Hymenoscyphus albidus* was never isolated from living tissue and this could cast doubts on the possibility of this species leading an endophytic lifestyle as suggested by Baral and Bemmann.” The current study, to the best of our knowledge, is the first one to record the natural colonization of ash leaves by *H. albidus* while the leaves are still attached to the tree. We did not attempt to establish Koch’s postulates for the necrotic symptoms associated with *H. albidus*, but inoculation of European ash rachis by *H. albidus* has been shown to induce short necrotic lesions (Kowalski et al., 2015).

The first necrotic leaflet veins associated with *H. albidus* in the stand in central Norway free of ash dieback were observed in late September or early October during the two sampling seasons. Like *H. albidus*, *H. fraxineus* is a harmless ash leaf associate in its native range, showing biomass increase in senescent, apparently asymptomatic ash leaves just before defoliation (Inoue et al., 2019), having an obvious capacity to induce leaf necrosis, as high levels of its DNA were recorded in necrotic leaflet lesions in Russia Far East in climatic autumn (Drenkhan et al., 2017). Thus, both fungi show a prolonged endophytic phase in ash leaves in their respective native range but can enter a necrotrophic growth phase in weakened host tissues. At an epidemic stage of ash dieback in the stand in southeastern Norway, the first *H. fraxineus*-associated necrotic lesions in ash leaves typically appear during late July/early August (Steinböck, 2013; Cross et al., 2017; Agan et al., 2020). Since the sporulation of *H. fraxineus* at this stand initiates in June and peaks between mid-July and mid-August (Hietala et al., 2013), the asymptomatic colonization phase in leaves of European ash is shorter for *H. fraxineus* than for *H. albidus*, which shows a similar sporulation period in Norway (Hietala et al., 2018b).

*Hymenoscyphus fraxineus* DNA level profiling by qPCR and high throughput sequencing of the ITS2 region at 1-week time intervals across a season in the Norderås site indicated a continuous accumulation of pathogen biomass in ash leaflets from the initiation of sporulation to mid-August when the peak sporulation period ends (Cross et al., 2017). In that study, the largest increments in pathogen DNA level between consequent samplings took place between the end of July and mid-August, a period during which symptoms of necrotic leaf lesions appeared. This data suggests that a certain threshold inoculum/tissue colonization level is needed for *H. fraxineus* to induce necrosis in leaf tissues and that once this level is reached, the pathogen biomass increases significantly. We are not aware of any study that would have considered the relation between inoculum level and induction of necrosis for *H. fraxineus*. By using a concentrated ascospore suspension (5 × 10^4^ ml^–1^) of *H. fraxineus*, Mansfield et al. (2019) showed that up to three ash leaf cells were colonized endophytically by the fungus before any plant cell death occurred, and the first lesions were observed under the inoculum droplet 7 days after inoculation. It seems reasonable to propose that the high infection pressure by *H. fraxineus* in comparison to *H. albidus* in European ash forests (Hietala et al., 2018b) allows the invader to switch from endophytic to necrotrophic growth phase before autumn senescence in Europe. Lower virulence of *H. albidus* in comparison to *H. fraxineus* could be a contributing factor to the late appearance of lesions associated with *H. albidus*, as leaf rachis inoculation of European ash by *H. albidus* produced shorter necrotic lesions than inoculation by *H. fraxineus* (Kowalski et al., 2015). Recently, Nielsen et al. (2022) proposed that there may be a positive relation between susceptibility to leaf and shoot infection by *H. fraxineus* – whether early induction of leaf necrosis could accelerate hyphal spread of the pathogen from leaves to shoots remains to be examined.

### The Role of Fecundity in the Ecology of *H. albidus* and *H. fraxineus*

At the inspected Stjørdal site, the formation of *H. albidus* fruiting bodies on ash leaf petioles on the forest floor was sparse during the period 2013–2017, when we monitored the stand – to the extent that it took some searching to find a petiole with fruiting bodies (unpublished observation). This is consistent with the general view that *H. albidus* is a rare species (Baral and Bemmann, 2014). In the Norderås site, as many as 10,000 *H. fraxineus* ascomata were found per m^2^ forest floor (Hietala et al., 2013), whereas it is difficult to conclude from the existing literature how prevalent the ascomata of *H. fraxineus* are in its native range. The native host Manchurian ash is highly susceptible to leaf infection by *H. fraxineus* (Nielsen et al., 2022), appears to support equally well ascomata production by *H. fraxineus* as European ash does (Nielsen et al., 2017), and it can also suffer from shoot dieback (Pastirčáková et al., 2020), a condition not reported from Asia.

Low ascomata production by *H. albidus* in comparison to *H. fraxineus*, both in field and laboratory conditions, prompted Wey et al. (2016) to propose that *H. albidus* may simply have inherently low fecundity. The low production of fruiting bodies and the fact that *H. albidus* is homothallic (Brasier et al., 2017) would suggest a *K*-selected strategy typical to stable environmental conditions and characterized by investment in fewer offspring, each of which has a relatively high fitness. The population size of *K*-selected species normally does not exceed the carrying capacity of the environment (MacArthur and Wilson, 1967; Pianka, 1970). In contrast, the high fecundity of *H. fraxineus*, production of recombinant offspring (Gross et al., 2012), and ability to survive as pseudosclerotia for several years before fruiting body formation (Kirisits, 2015) may suggest that the lineage of *H. fraxineus* invasive in Europe has been subjected to *r*-selection in its native range, a strategy representing an adaptation to harsh and unstable environmental conditions (Pianka, 1970). The native hosts of *H. fraxineus* span multiple climate zones in Asia, these including regions with cold, dry winters and either hot, warm, or cold summers (Wei et al., 2004; Peel et al., 2007). However, the origin of the *H. fraxineus* lineage invasive in Europe remains unknown as the ITS rDNA sequence variant of *H. fraxineus* predominant in Europe is very rare in the so far explored regions in Asia (Drenkhan et al., 2017). European ash and Manchurian ash, the native hosts of *H. albidus* and *H. fraxineus*, belong to the section *Fraxinus* (Wallander, 2012) which includes black ash (*Fraxinus nigra*) and narrow-leafed ash (*Fraxinus angustifolia*): all four are highly susceptible to leaf infection by *H. fraxineus*, indicating the presence of a phylogenetic signal in susceptibility (Nielsen et al., 2017). It is possible that the climatic conditions prevailing throughout most of the distribution range of European ash may be more favorable to *H. fraxineus* life cycle completion than the conditions in its native range, this tipping the balance between the host and a fungus that is essentially an endophyte.

There are very few other studies available that have compared the behavior of invasive fungal tree pathogens and their native relatives. When monitoring the sporulation of *Heterobasidion irregulare*, a North American conifer pathogen introduced to central Italy, Garbelotto et al. (2010) concluded that the superior reproductive capacity of this invader in dry summer seasons in comparison to *H. annosum*, a native competitor, facilitates the invasiveness of this fungus. Like us, Garbelotto et al. (2010) proposed that the high spore production capacity of *H. irregulare* may be a trait present in the founding population.

### Hyphal Affinity to Different Cells in Leaflets Colonized by *H. albidus* or *H. fraxineus*

When considering our histological observations, it should be kept in mind that the studied materials represent natural infection of multiple fungal species, and that the vegetative hyphae of *H. fraxineus* and *H. albidus* do not possess any specific features that would allow their indisputable identification. On agar, the hyphal diameter of the type-strain of *H. fraxineus* ranged between 1.2 and 4.2 μm (Kowalski, 2006), and in ash shoots, upon inoculation trials, hyphae in the diameter range between 1.1 μm (Dal Maso et al., 2012) and 7 μm (Schumacher et al., 2010) have been associated to *H. fraxineus.* No such literature references seem to be available for *H. albidus*. The fungal hyphae now detected in leaf tissues, hosting as much as >200 pg of either *H. albidus* or *H. fraxineus* DNA per mm^2^ tissue, had diameter in the range between 2 and 6 μm. Assuming that the propagules of these fungi are predominantly uninucleate *in planta*, this level would translate to above 3,000 fungal cells/mm^2^ leaf tissue as the estimated 62 MB haploid genome size of *H. fraxineus* (McMullan et al., 2018) and 51.2 MB haploid genome size of *H. albidus* (Elfstrand et al., 2021) correspond to 0.0634 and 0.0530 pg DNA, respectively.

When comparing fungal colonization patterns between leaflets hosting a high level of either *H. albidus* or *H. fraxineus* DNA, there was no obvious difference in the host cell types the fungal hyphae were associated with. In both cases, one prominent feature was the high colonization of sclerenchyma and phloem, a region from which the ascomata of both species arise (Baral and Bemmann, 2014). In addition, fungal hyphae showed high affinity to axial parenchyma, cells that are rich in starch in the growing season (Nielsen et al., 2022). The affinity of *H. fraxineus* to parenchyma cells has been observed both in ash shoots (Schumacher et al., 2010; Dal Maso et al., 2012; Matsiakh et al., 2016) and leaves (Nielsen et al., 2022). The generally low level of starch detected in the present material is obviously partly due to the late season as the samples were collected in September or October. Another factor that may have contributed to the low level of starch is either its conversion to defense phenolics or metabolization by fungi.

### Fungal Community Structure in Healthy and Diseased Forests and Community Equilibrium

The number of fungal OTUs detected in leaflet samples was by far highest for the Norderås site with an epidemic level of ash dieback, followed by the ash dieback affected Vedu site and the healthy Stjørdal site, with an average of 52, 31, and 19 OTUs, respectively. However, the size of tissue processed differed correspondingly: entire leaflets were analyzed for Norderås, whereas for Vedu and Stjørdal the processed leaflet subsamples had an area of 100 and 12 mm^2^, respectively. Data comparison across different sampling schemes is complicated because fungal composition may differ between veinal and interveinal regions of ash leaflets owing to differences in microenvironment quality and fungal preferences for different microsites, as discussed by Agan et al. (2020). As illustrated by our separate analysis of necrotic and green tissues from Stjørdal, fungal species composition may depend on tissue condition as well, something that will go undetected when analyzing large samples composed of both healthy and necrotic tissues. None of the prior studies describing fungal community structure in ash leaf tissues in stands infested by *H. fraxineus* considered separately necrotic and asymptomatic tissues (Cross et al., 2017; Schlegel et al., 2018; Griffiths et al., 2019; Agan et al., 2020).

By using a qPCR assay specific to *H. fraxineus* DNA, Steinböck (2013) recorded high levels of *H. fraxineus* DNA in necrotic leaflet veins, this being confirmed in the present study. However, more noteworthy is our observation that also the indigenous *H. albidus* showed a high DNA level and sequence read proportion within necrotic tissues in leaflets collected in late autumn in a stand with no shoot dieback symptoms. The material analyzed for *H. albidus* colonized leaflets is small, because symptomatic leaflets were rare in the healthy stand, even in late autumn. In common with findings of metabarcoding studies from stands with ash dieback (Cross et al., 2017; Schlegel et al., 2018; Griffiths et al., 2019; Agan et al., 2020), the leaflets in the healthy stand showed the prevalence of ascomycetous endophytes with pathogenic potential in the genera *Venturia* (and related *Fusicladium* anamorphs), *Mycosphaerella* (and related *Ramularia* anamorphs), *Phoma* and *Cladosporium* – as a group, endophytic fungi with pathogenic potential showed higher sequence read proportions in necrotic tissue areas than in green tissues. There are very few studies available on leaf microbiomes of European ash from healthy forests, but species of *Venturia*, *Ramularia*, *Phoma*, and *Cladosporium* were frequently isolated from surface-sterilized healthy ash leaves in a German site free of ash dieback (Reiher, 2011). Like *Hymenoscyphus* species, *Venturia*, *Mycosphaerella*, and *Ramularia* species are considered to show high host specificity (Videira et al., 2016; González-Domínguez et al., 2017).

It is noteworthy that the sequence read proportions of *Ramularia* spp. correlated positively with those of *H. albidus* in the present study and with those of *H. fraxineus* in the Vedu site in our prior study (Agan et al., 2020). *Ramularia* anamorphs and related *Mycosphaerella* species were the most common fungi detected in healthy leaves of Manchurian ash in Far East Russia (Cleary et al., 2016). Like these *Hymenoscyphus* species, many *Ramularia* species also exhibit a prolonged endophytic growth stage before switching to the necrotrophic phase late in the growing season upon changes in host physiological condition (McGrann et al., 2016). Whether common host cues promote the switch of *H. albidus*, *H. fraxineus*, and ash associated *Ramularia* species from endophytic to necrotrophic growth phase remains to be studied.

The holobiont theory sees microbiomes as extensions of host phenotype (Agler et al., 2016) – to understand the invasive success of *H. fraxineus*, one needs to also consider the resident microbial communities associated with ash. The “Diversity Resistance” hypothesis argues that diverse communities are highly competitive and readily resist invasion based on the assumption that niche space in diverse natural communities is a limiting factor, and that such communities are structured by interspecific competition (Levine and D’Antonio, 1999; Laforest-Lapointe et al., 2017). A significant proportion of endophytic fungi (80%) produce antibacterial and fungicidal compounds (Schulz et al., 2002), this applies to *Hymenoscyphus fraxineus* and *H. albidus* as well (Halecker et al., 2014, 2017; Junker et al., 2014). Reciprocal antagonism is a common reaction between colonies of *H. fraxineus* and endophytes of common ash (Junker, 2013; Schulz et al., 2015; Haňáčková et al., 2017a) on agar conditions. Exudates from several ash endophytes can also suppress the germination of *H. fraxineus* ascospores (Schlegel et al., 2016). However, such tests do not take into account the sharp differences in the *in planta* biomass between individual endophyte species and *H. fraxineus*. The biomass of *H. fraxineus* at the stage of leaf necrosis formation – owing to feedback from the saprobic stage – obviously exceeds that of any resident endophyte, present presumably as scattered small thalli. This asymmetry may be advantageous to *H. fraxineus* in species race for the capture of weakened host tissues. As far as we know, no confrontation studies have yet been carried out between colonies of *H. albidus* and other endophytes of ash leaves. One could envisage that *H. albidus* may be more subject to control by co-inhabiting endophytes than *H. fraxineus* upon the fungal race following leaf tissue weakening owing to its low propagule pressure.

## Conclusion

A noteworthy common feature of *H. albidus* and *H. fraxineus* is that in their native range, they appear as harmless endophytes and saprophytes in leaf tissues of their co-evolved hosts but can show some capacity to enter a necrotrophic growth phase in late autumn. This suggests a link between host physiological condition and the colonization mode of these fungi. In Europe, the most striking difference between the two species appears to be their differential fecundity. *H. albidus* has features of *K*-selected species and the lineage of *H. fraxineus* invasive in Europe those of *r*-selected species, suggesting that they have been subjected to different environmental selection pressures in their native range. The adaptation to a host that is phylogenetically closely related to European ash, a tree species with high occurrence frequency in Europe, and the presence of environmental conditions favorable to *H. fraxineus* life cycle completion in most years may enable the build-up of high infection pressure and challenge of leaf defense responses already during the growing season.

## Data Availability Statement

The datasets presented in this study can be found in online repositories. Filtered representative sequences for each sample from Stjørdal are available in PlutoF—location: https://dx.doi.org/10.15156/BIO/2483903. All raw sequences from Stjørdal are available in SRA (sequence read archive) under accession number: PRJNA808236. Filtered representative sequences from Vedu and Norderås sites are available in PlutoF—location: https://dx.doi.org/10.15156/BIO/2483893. Raw sequences from Vedu and Norderås sites are available in SRA under accession number: PRJNA638044.

## Author Contributions

AH, IB, NN, HS, and VT collected the study material. AH did the qPCR analyses. NN did the histochemical analyses. AA processed the DNA samples to sequencing and did the bioinformatics analyses. AH and AA drafted the manuscript. All authors participated in the designing of the study, data interpretation, and editing of the final manuscript, and approved the submitted version.

## Conflict of Interest

The authors declare that the research was conducted in the absence of any commercial or financial relationships that could be construed as a potential conflict of interest.

## Publisher’s Note

All claims expressed in this article are solely those of the authors and do not necessarily represent those of their affiliated organizations, or those of the publisher, the editors and the reviewers. Any product that may be evaluated in this article, or claim that may be made by its manufacturer, is not guaranteed or endorsed by the publisher.
