# Supplementary material for: The Native Hymenoscyphus albidus and the Invasive Hymenoscyphus fraxineus Are Similar in Their Necrotrophic Growth Phase in Ash Leaves
Source: Front Microbiol. 2022 May 27;13:892051. doi: 10.3389/fmicb.2022.892051 (PMC9196304; doi:10.3389/fmicb.2022.892051)
Supplement: Supplementary file 1 [file Table_1.DOCX]

| **Supplementary Table S1.** Abundance, growth form in vegetative phase and hypothetical functional group of fungal species detected on leaflets of *Fraxinus excelsior* in Stjørdal, Norderås and Vedu*.* Growth form: F, filamentous; Y, yeast; D, dimorphic. Hypothetical functional group: En/N, endophytic with some capacity to cause necrosis; Ep/En can occur both as an epiphyte and an endophyte; B, biotrophic. The putative functional groups are deduced from literature, often from another host than *Fraxinus* and thus there is uncertainty about this. | | | | | |
| --- | --- | --- | --- | --- | --- |
| **Site** | **Growth form** | **Hypothetical functional group** | **OTU assignment** | **UNITE species hypotheses** | **Relative abundance %** |
| Stjørdal | F | En/N | *Hymenoscyphus albidus* | SH1543028.08FU | 21.57 |
|  | ? | ? | Fungi sp. | SH1647619.08FU | 6.01 |
|  | F | En/N | *Phoma* sp. | SH1547057.08FU | 5.70 |
|  | Y | Ep/En | Tremellomycetes sp. | SH1528209.08FU | 4.78 |
|  | Y | Ep/En | Bulleribasidiaceae sp. | SH1609773.08FU | 4.62 |
|  | F | En/N | *Fusicladium proteae* | SH1626889.08FU | 4.16 |
|  | F | En/N | *Mycosphaerella punctiformis* | SH1577460.08FU | 4.16 |
|  | F | En/N | *Venturia fraxini* | SH1626889.08FU | 3.85 |
|  | F | En/N | *Ramularia phacae-frigidae* | SH1577460.08FU | 3.39 |
|  | Y | Ep/En | *Dioszegia* sp1 | SH1211794.08FU | 3.39 |
|  | Y | Ep/En | *Vishniacozyma victoriae*1 | SH1528207.08FU | 3.24 |
|  | F | En/N | *Cladosporium* sp. | SH1572792.08FU | 1.85 |
|  | Y | Ep/En | *Vishniacozyma victoriae*2 | SH1528207.08FU | 1.69 |
|  | ? | ? | Fungi sp. | SH1648647.08FU | 1.69 |
|  | F | ? | Leptosphaeriaceae sp. | SH1614481.08FU | 1.54 |
|  | D | ? | *Tilletiopsis* sp. | SH1539625.08FU | 1.39 |
|  | D | B | *Taphrina* sp. | SH1519932.08FU | 1.39 |
|  | Y | Ep/En | *Bullera crocea* | SH1609773.08FU | 1.39 |
|  | Y | Ep/En | *Dioszegia* sp2 | SH1609794.08FU | 1.23 |
|  | F | En/N | *Ramularia pratensis* | SH1577492.08FU | 1.23 |
| Norderås | F | En/N | *Hymenoscyphus fraxineus* | SH1543028.08FU | 20.04 |
|  | Y | Ep/En | *Dioszegia* sp. | SH1609794.08FU | 7.70 |
|  | F | B | *Phyllactinia fraxini* | SH1528127.08FU | 7.66 |
|  | Y | Ep/En | *Vishniacozyma heimaeyensis*1 | SH1528210.08FU | 4.23 |
|  | Y | Ep/En | *Filobasidium wieringae* | SH1631613.08FU | 3.67 |
|  | Y | Ep/En | *Vishniacozyma carnescens* | SH1528208.08FU | 3.47 |
|  | F | En/N | *Phoma* sp. | SH1547057.08FU | 3.31 |
|  | ? | ? | Fungi sp. | SH1572820.08FU | 3.03 |
|  | F | B | *Exobasidium gracile* | SH1528190.08FU | 2.28 |
|  | F | En/N | *Venturia* sp. | SH1626893.08FU | 2.08 |
|  | Y | Ep/En | *Vishniacozyma victoriae* | SH1528207.08FU | 2.04 |
|  | Y | Ep/En | *Vishniacozyma heimaeyensis* 2 | SH1528210.08FU | 1.88 |
|  | D | B | *Taphrina padi* | SH1519937.08FU | 1.84 |
|  | ? | ? | Fungi sp. | SH2725593.08FU | 1.76 |
|  | F | En/N | *Cladosporium* sp. | SH1572792.08FU | 1.52 |
|  | Y | Ep/En | *Papiliotrema flavescens* | SH1576891.08FU | 1.52 |
|  | Y | Ep/En | *Vishniacozyma* sp. | SH1528208.08FU | 1.36 |
|  | F | En/N | *Phoma* sp. | SH1547057.08FU | 1.28 |
|  | Y | Ep/En | Tremellomycetes sp. | SH1528209.08FU | 1.24 |
|  | F | En/N | *Mycosphaerella punctiformis* | SH1577460.08FU | 1.08 |
| Vedu | F | En/N | *Hymenoscyphus fraxineus* | SH1543028.08FU | 29.35 |
|  | F | En/N | *Venturia fraxini*1 | SH1626889.08FU | 13.21 |
|  | F | En/N | *Fusicladium proteae*1 | SH1626889.08FU | 7.83 |
|  | F | En/N | *Phoma* sp.1 | SH1547057.08FU | 5.01 |
|  | D | Ep/En | *Aureobasidium* sp. | SH1515060.08FU | 4.90 |
|  | F | En/N | *Cladosporium* sp. | SH1572792.08FU | 4.71 |
|  | Y | Ep/En | *Dioszegia* sp. | SH1609794.08FU | 4.34 |
|  | Y | Ep/En | *Kondoa* sp. | SH1566866.08FU | 2.67 |
|  | Y | Ep/En | *Vishniacozyma carnescens* | SH1528208.08FU | 2.08 |
|  | ? | ? | Fungi sp. | SH1572820.08FU | 1.67 |
|  | F | B | *Phyllactinia fraxini* | SH1528127.08FU | 1.22 |
|  | F | En/N | *Phoma* sp2 | SH1547057.08FU | 1.22 |
|  | D | Ep/En | *Aureobasidium pullulans* | SH1515060.08FU | 1.11 |
|  | Y | Ep/En | *Vishniacozyma heimaeyensis* | SH1528210.08FU | 1.00 |
|  | F | En/N | *Venturia* sp. | SH1626893.08FU | 0.63 |
|  | F | En/N | *Venturia fraxini* 2 | SH1626889.08FU | 0.63 |
|  | ? | ? | Fungi sp. | SH1525140.08FU | 0.56 |
|  | F | En/N | *Fusicladium proteae*2 | SH1626889.08FU | 0.56 |
|  | Y | Ep/En | *Bullera alba* | SH1574527.08FU | 0.52 |
|  | F | En/N | *Venturia fraxini* 3 | SH1626889.08FU | 0.41 |
|  |  |  |  |  |  |
