# Supplementary material for: The Native Hymenoscyphus albidus and the Invasive Hymenoscyphus fraxineus Are Similar in Their Necrotrophic Growth Phase in Ash Leaves
Source: Front Microbiol. 2022 May 27;13:892051. doi: 10.3389/fmicb.2022.892051 (PMC9196304; doi:10.3389/fmicb.2022.892051)
Supplement: Supplementary file 2 [file Data_Sheet_1.PDF]

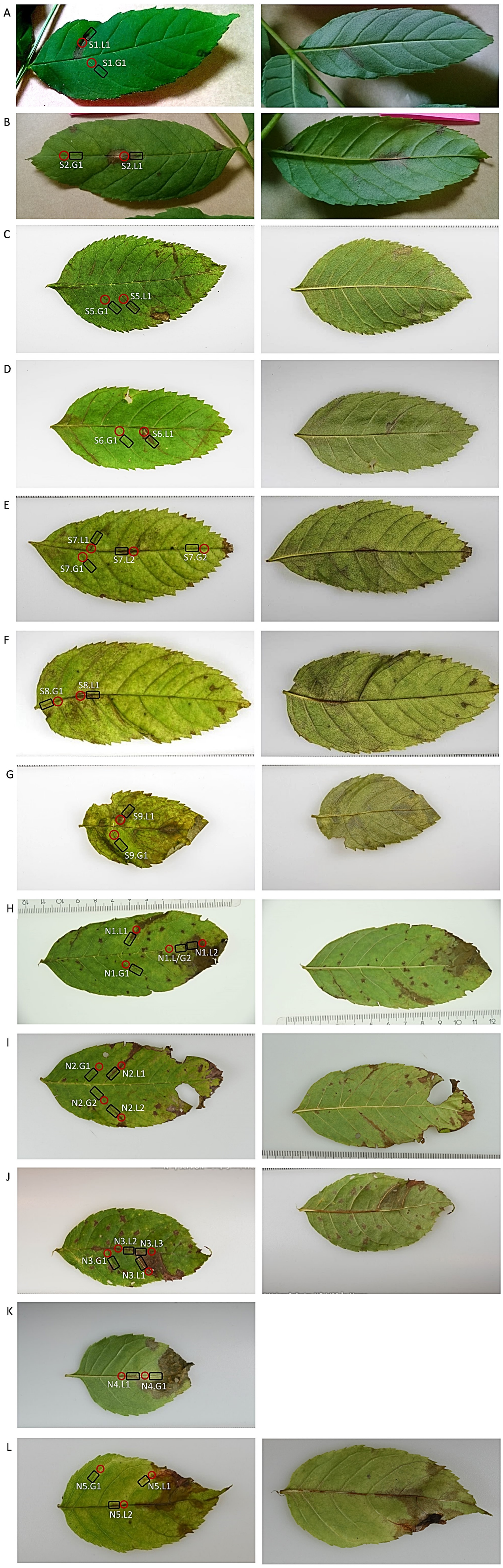

Supplementary Figure 1. Images of leaflet samples collected from the two forest stands in Norway 2016-2017, **(A-G)** Stjørdal and **(H-L)** Norderås. The adaxial domain of the leaflet to the right, and the corresponding abaxial domain to the left. Red circle indicates subsamples dissected for DNA analyses, and black squares areas dissected for microscopy. Samples collected were including tissue both from lesion areas and from green areas without lesion from all leaflets. Abbreviations: S = Stjørdal, N = Norderås, L = Lesion, G = Green.
